# *Impatiens lianchengensis* (Balsaminaceae), a new species from southeastern China

**DOI:** 10.3897/phytokeys.280.199638

**Published:** 2026-10-15

**Authors:** Jiedong Lin, Junlong Li, Boheng Li, Xinjie Jin, Meizhen Wang, Pan Li

**Affiliations:** 1 College of Biology and Environmental Engineering, Zhejiang Shuren University, Hangzhou 310015, China College of Biology and Environmental Engineering, Zhejiang Shuren University Hangzhou China; 2 Educational Technology Center of Fujian Province, Fuzhou 350003, China Educational Technology Center of Fujian Province Fuzhou China; 3 College of Life Science, Zhejiang Sci-Tech University, Hangzhou 330104, China College of Life Science, Zhejiang Sci-Tech University Hangzhou China; 4 YOUNG NATURALIST (Hangzhou) Cultural Communication Co., Ltd., Hangzhou 310058, China YOUNG NATURALIST (Hangzhou) Cultural Communication Co., Ltd. Hangzhou China; 5 College of Life and Environmental Science, Wenzhou University, Wenzhou 325035, China College of Life and Environmental Science, Wenzhou University Wenzhou China; 6 Key Laboratory of Biodiversity and Environment on the Qinghai-Tibetan Plateau, Ministry of Education, School of Ecology and Environment, Xizang University, Lhasa 850000, China Key Laboratory of Biodiversity and Environment on the Qinghai-Tibetan Plateau, Ministry of Education, School of Ecology and Environment, Xizang University Lhasa China; 7 Motuo Biodiversity Observation and Research Station of Xizang Autonomous Region, Motuo 860700, China Motuo Biodiversity Observation and Research Station of Xizang Autonomous Region Motuo China

**Keywords:** Balsaminaceae, *Impatiens lianchengensis*, phylogeny, taxonomy

## Abstract

A new species, *Impatiens lianchengensis* J. D. Lin & P. Li, **sp. nov**., from a moist valley on Mt. Guanzhai, Liancheng County, Fujian Province, southeastern China, is described and illustrated. It resembles *I. huangyanensis* X. F. Jin & B. Y. Ding and *I. tainingensis* J.-D. Lin & P. Li in gross morphology but differs in numerous features. The molecular phylogeny of *Impatiens* indicated that *I. lianchengensis* is closely related to *I. kuocangshanica*, *I. wuyiensis*, *I. platysepala*, and *I. tainingensis* based on plastome and ITS sequences.

## Introduction

The family Balsaminaceae, belonging to the order Ericales, comprises two genera: *Hydrocera* Blume ex Wight & Arn. and *Impatiens* Riv. ex L. (Richard 2000). *Hydrocera* contains only one species, while *Impatiens* has over 1000 species distributed in paleotropical and subtropical regions (Grey-Wilson 1980; Fischer and Rahelivololona 2004; Yu et al. 2016). China is recognized as one of the five global diversity hotspots for *Impatiens*, harboring approximately 352 species (273 of them endemic), most of which are concentrated in the mountainous regions of southwestern China (Chen et al. 2007; Yu 2012; Yuan et al. 2022; Chen et al. 2023).

As J. D. Hooker noted (Hooker 1908), *Impatiens* is one of the most taxonomically challenging groups of flowering plants. Its fleshy stems and thin, translucent perianth make specimens fragile and easily deformed, making floral structures difficult to restore once dried. Given these challenges, field-based investigations and regional floristic surveys are crucial for clarifying species boundaries and distribution patterns. In recent years, new *Impatiens* species have been published mainly from southwestern China, such as Guangxi and Xizang (Du et al. 2021). In eastern China, Danxia landforms are also found in Zhejiang and Fujian, where *Impatiens* species are distributed and endemism is common (Peng et al. 2018). Three new endemic species have recently been described in Fujian Province, *I. wuyiensis* J.S.Wang, Y.F.Lu & X.F.Jin (Wang et al. 2020), *I. fujianensis* Liang Ma, Xin Y.Chen & S.P.Chen (Ma et al. 2024), and *I. tainingensis* J.-D. Lin & P. Li (Wang et al. 2026), further indicating that the diversity of *Impatiens* in this region has not yet been fully revealed.

During a survey in Fujian Province, a suspected new *Impatiens* species was found in a valley of Guanzhaishan, Liancheng County. It appeared to be similar to *I. huangyanensis* and *I. tainingensis* in the gross morphology of their pinkish purple flowers but differed in its leaves, inflorescences, flowers, and capsules. Molecular phylogeny indicates that it is closely related to *I. tainingensis* and *I. platysepala*, further confirming the distinct nature of this putative new species. It is therefore recognized as a new species, *Impatiens lianchengensis* J. D. Lin & P. Li, sp. nov., and is described and illustrated here.

## Materials and methods

### Sampling and morphology

In 2012 and 2022, several field excursions were made to Liancheng County in Fujian Province to collect and observe this species. The morphological characters were recorded and measured based on living plants and specimens. Herbarium specimens from CDBI, CSH, FUN, HHBG, HIB, HZU, IBSC, KUN, LBG, NAS, PE, and WUK (acronyms according to Index Herbariorum, https://sweetgum.nybg.org/science/ih/) were also examined for morphological comparison. Voucher specimens were deposited at the Herbarium of Zhejiang University (HZU, acronym according to Index Herbariorum, https://sweetgum.nybg.org/science/ih/).

### DNA sequencing and phylogenetic analyses

Two accessions were sequenced for molecular analyses, and both individuals were collected from the same known population at Mt. Guanzhai (Suppl. material 1). Genomic DNA was extracted from silica gel-dried leaf tissues using Plant DNAzol reagent (Shanghai, China) following the manufacturer’s instructions. Sequencing libraries were constructed at the China National GenBank (CNGB, Shenzhen, China), and 150 bp paired-end reads were generated on the Illumina HiSeq X10 platform. To infer the phylogenetic position of the species, both the nuclear ribosomal ITS region and the complete plastome sequence were analyzed. All newly obtained sequences were deposited in GenBank (Suppl. material 2). The plastome and ITS sequences were assembled using GetOrganelle (Jin et al. 2020) under default parameters, and sequence alignments were performed with MAFFT v. 7.487 (Katoh and Standley 2013). Annotation of the plastome was performed using PGA software (Qu et al. 2019), and the circular structure of ptDNA was drawn using Organellar Genome DRAW (Lohse et al. 2007). Maximum-likelihood (ML) phylogenetic analyses were conducted in IQ-TREE 2 (Minh et al. 2020) with 1000 bootstrap replicates, and optimal substitution models were selected by ModelFinder (Kalyaanamoorthy et al. 2017) for each partition. *Hydrocera triflora* was designated as the outgroup. The resulting ML tree was visualized and edited using FigTree v. 1.4.4 (http://tree.bio.ed.ac.uk/software/figtree/).

## Results

### Morphological comparison

**Figure 1.**
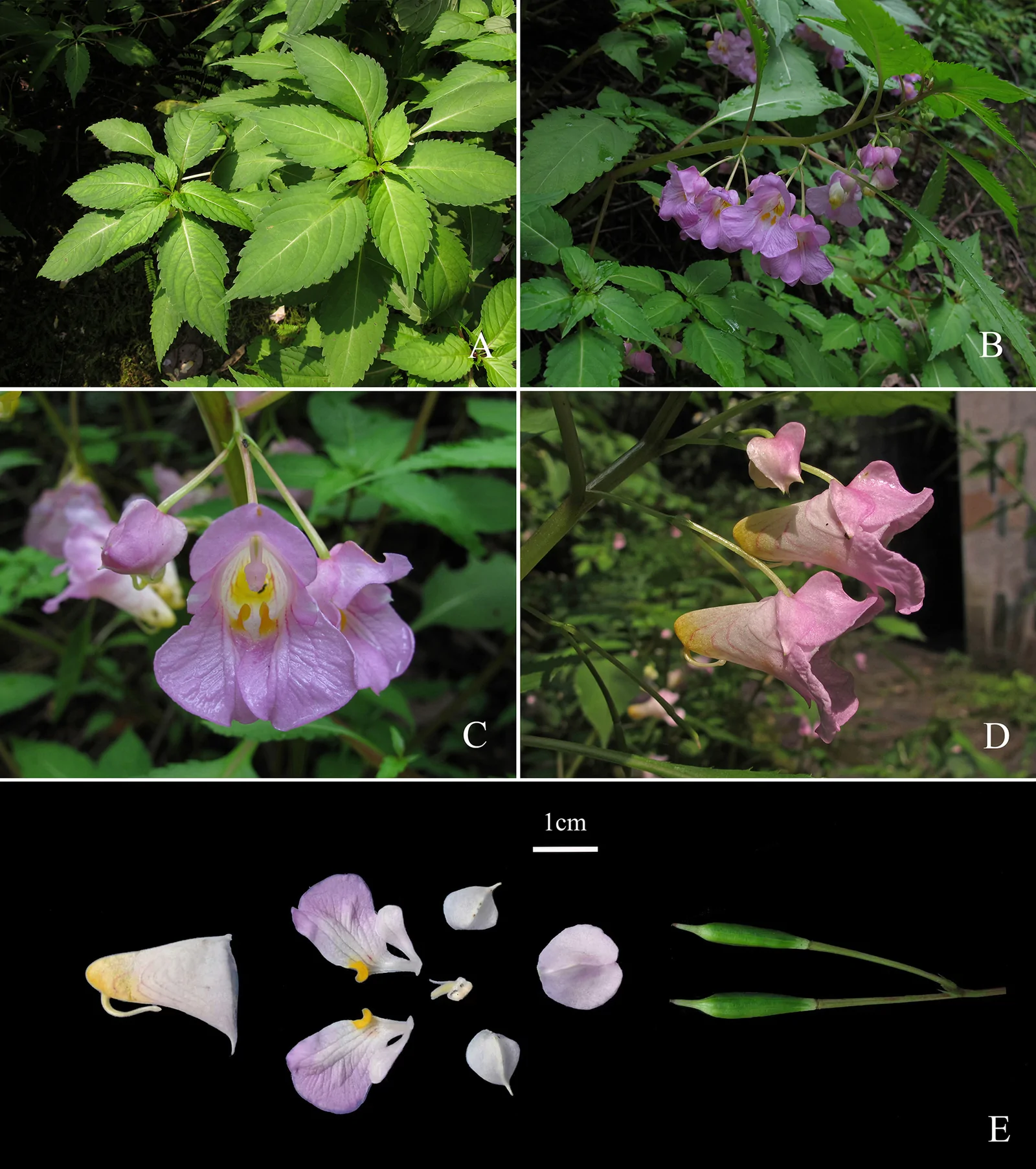
*Impatiens lianchengensis*. **A**. Plant; **B**. Flowering plant; **C**. Flower, front view; **D**. Flower, lateral view; **E**. Dissection of flower and capsule [Photos by Jie-Dong Lin].

**Figure 2.**
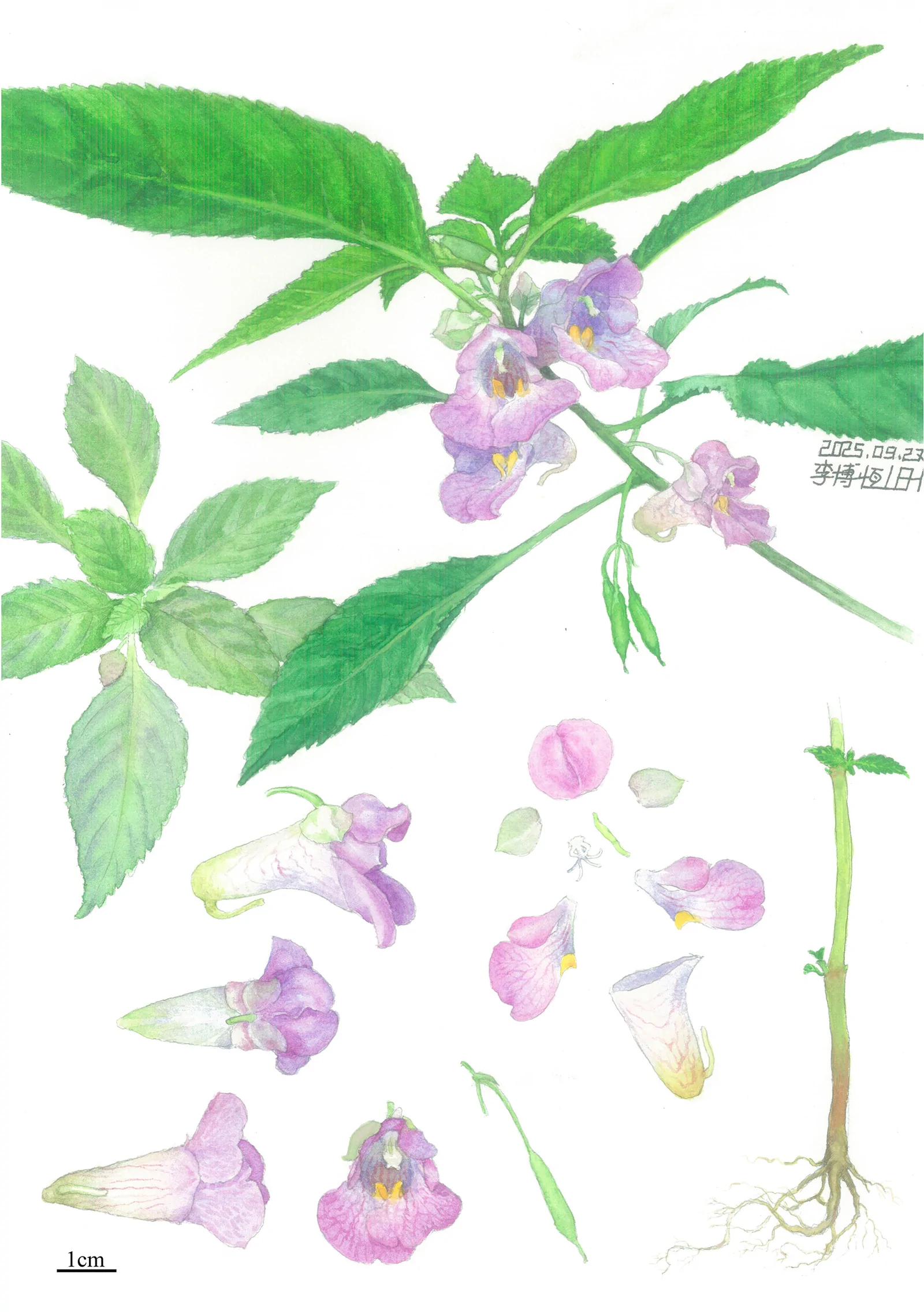
Painted illustration of *Impatiens lianchengensis* [Drawn by Bo-Heng Li].

**Figure 3.**
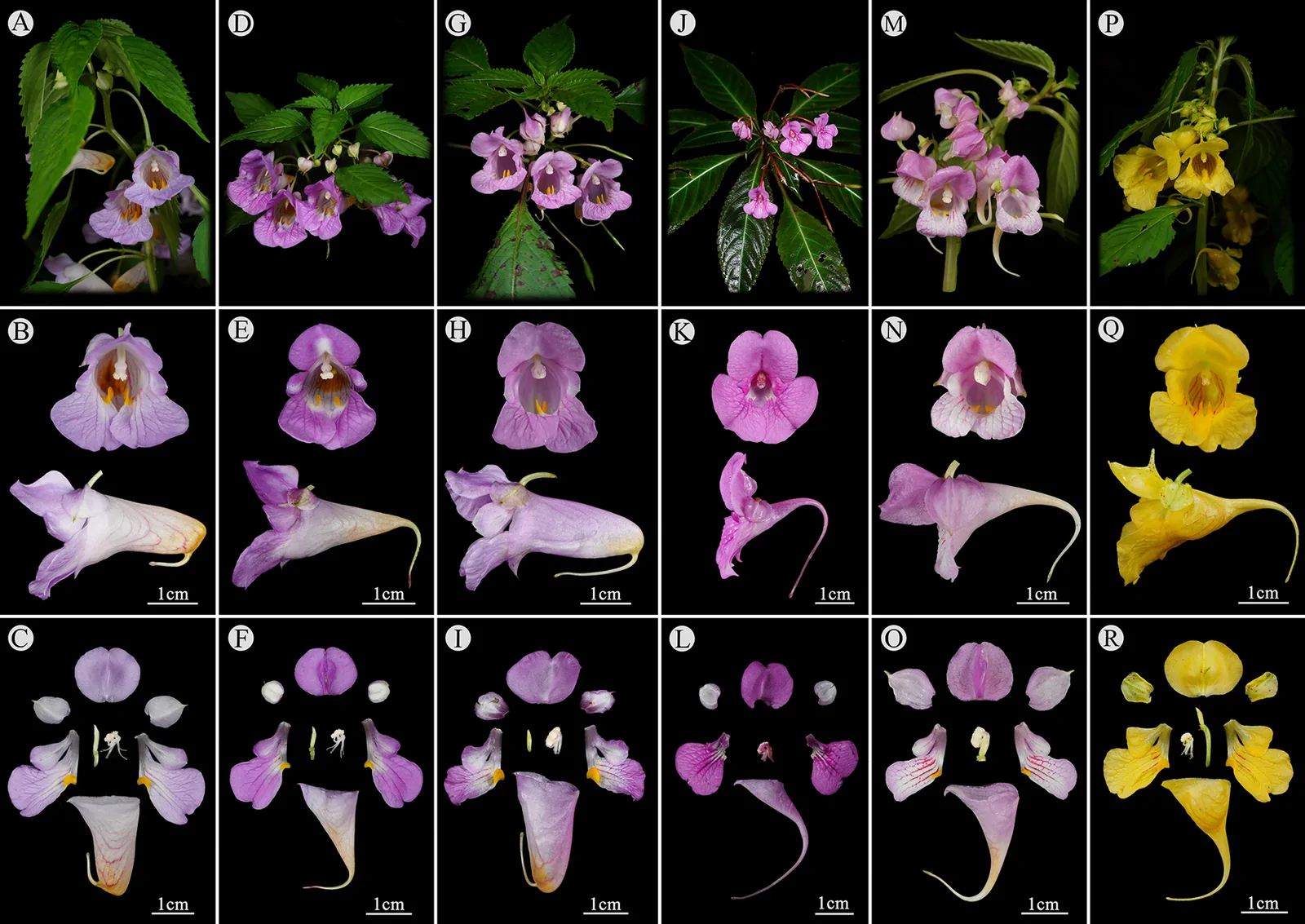
Morphological comparison of *Impatiens lianchengensis* (**A–C**); *I. tainingensis* (**D–F**); *I. huangyanensis* (**G–I**); *I. chekiangensis* (**J–L**); *I. kuocangshanica* (**M–O**); and *I. wuyiensis* (**P–R**) [Photos by Jun-Long Li].

**Figure 4.**
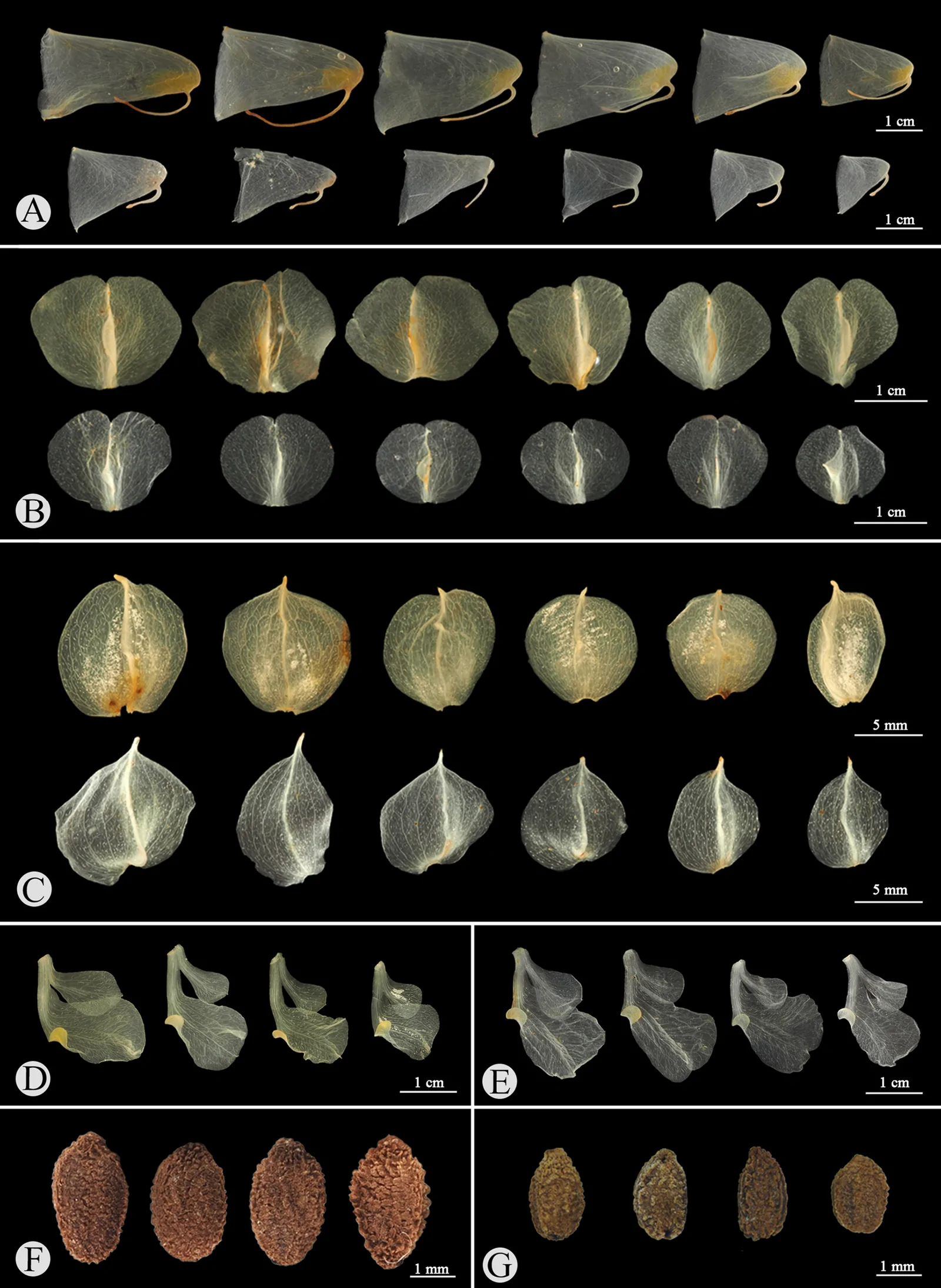
Comparison of floral and seed morphology between *Impatiens huangyanensis* and *I. lianchengensis*. **A**. Comparison of lower sepals (labellum). Top row: *I. huangyanensis*; bottom row: *I. lianchengensis*; **B**. Comparison of upper petals. Top row: *I. huangyanensis*; bottom row: *I. lianchengensis*; **C**. Comparison of lateral sepals. Top row: *I. huangyanensis*; bottom row: *I. lianchengensis*; **D**. Lateral united petals (wings) of *I. huangyanensis*; **E**. Lateral united petals (wings) of *I. lianchengensis*; **F**. Seeds of *I. huangyanensis*; **G**. Seeds of *I. lianchengensis*. Scale bars: 1 cm (**A, B, D, E**); 5 mm (**C**); 1 mm (**F, G**) [Photos by Jun-Long Li].

Detailed morphological comparisons among *Impatiens lianchengensis*, *I. huangyanensis*, and *I. tainingensis* are summarized in Table 1. *I. lianchengensis* (Figs 1, 2) resembles *I. huangyanensis* and *I. tainingensis* in the gross morphology of their pinkish purple flowers but can be distinguished by a combination of leaf and floral characters (Fig. 3). It differs from *I. tainingensis* in having a wider, more abruptly saccate lower sepal with an abruptly narrowed and shorter spur (5.6–13.0 mm vs. 15–18 mm). Among the quantitative characters examined, five were found to be of significant diagnostic value in distinguishing *I. huangyanensis* from *I. lianchengensis*. The spur length of *I. huangyanensis* ranged from 12.8 to 26.1 mm (mean 18.91 mm), whereas that of *I. lianchengensis* ranged from 5.6 to 13.0 mm (mean 9.44 mm), with only a slight overlap at 12.8–13.0 mm. The upper petal height was 14.6–17.8 mm (mean 15.96 mm) in *I. huangyanensis* and 10.8–13.7 mm (mean 12.47 mm) in *I. lianchengensis*; the upper petal width was 17.5–22.4 mm (mean 20.47 mm) in *I. huangyanensis* and 12.1–16.5 mm (mean 14.5 mm) in *I. lianchengensis*. The ranges of both upper petal dimensions were completely non-overlapping between the two species. Seeds also differed considerably: seed length was 2.74–3.11 mm (mean 2.89 mm) in *I. huangyanensis* and 1.8–2.2 mm (mean 2.01 mm) in *I. lianchengensis*; seed width was 1.75–2.12 mm (mean 1.92 mm) in *I. huangyanensis* and 1.1–1.3 mm (mean 1.18 mm) in *I. lianchengensis*, with completely non-overlapping ranges. In qualitative characters, *I. lianchengensis* has a widely saccate lower sepal, with a relatively wide mouth and a distinctly attenuate posterior end; its lateral sepals are obliquely ovate, and its upper petal is suborbicular. In contrast, *I. huangyanensis* has a saccate to widely saccate lower sepal, with the posterior end not distinctly attenuate; its lateral sepals are suborbicular, and its upper petal is oblate. The two species can be distinguished by these characters. Therefore, spur length, upper petal height, upper petal width, seed length, seed width, lower sepal shape, lateral sepal shape, and upper petal shape can serve as reliable diagnostic characters for distinguishing these two species (Fig. 4).

**Table 1.** Comparison between *Impatiens lianchengensis*, *I. tainingensis*, and *I. huangyanensis*.

**Characters**	***I. lianchengensis***	***I. tainingensis***	***I. huangyanensis***
Plant height	30–70 cm tall	40–80 cm tall	30–80 cm tall
Leaves	5–14 × 2–5 cm, lateral veins 5–8 pairs	5–11 × 2–3.5 cm, lateral veins 5–7 pairs	6–17 × 2–6 cm, lateral veins 6–8 pairs
Inflorescences	Flowers 1–5; peduncles 1–2 cm long; pedicels 2–3 cm long; bracts 2–3 mm long	Flowers 1–3; peduncles 1–2 cm long; pedicels 1.5–2.5 cm long; bracts 1–2 mm long	Flowers 1–6; peduncles 1 cm long; pedicels 1–2 cm long; bracts 3–6 mm long
Lateral sepals	Obliquely ovate, 8–11 × 6–9 mm	Suborbicular, 6–8 × 5–8 mm	Suborbicular, 7–10 × 8–9 mm
Lower sepal	12.2–23.4 mm deep, widely saccate, mouth relatively wide, posterior end distinctly attenuate, abruptly narrowed into an incurved spur apart at the bottom; spur 5.6–13.0 mm long; mouth vertical, 13.2–18.7 mm wide	19–24 mm deep, subsaccate-funnelform, gradually narrowed into an incurved spur; spur 15–18 mm long; mouth vertical, 15–17 mm wide	19.9–33.9 mm deep, saccate to widely saccate, posterior end not distinctly attenuate, abruptly narrowed into an incurved spur; spur 12.8–26.1 mm long; mouth vertical, 15.1–23.3 mm wide
Upper petal	Suborbicular, 10.8–13.7 × 12.1–16.5 mm	Oblate, 12–13 × 17–19 mm	Oblate, 14.6–17.8 × 17.5–22.4 mm
Lateral united petals	Basal lobes obovate to dolabriform, 10.9–12.7 × 4.5–6.9 mm; distal lobes 19.4–24.4 × 9.5–14.2 mm	Basal lobes obovate to dolabriform, 0.9–1.0 × 0.6–0.7 cm; distal lobes 2.0–2.2 × 1.2–1.4 cm	Basal lobes obovate, 11.0–15.2 × 5.2–8.3 mm; distal lobes 18.5–25.4 × 8.8–14.0 mm
Capsule	16.7–25.0 mm long	15–21 mm long	18.6–29.6 mm long
Seeds	1.8–2.2 × 1.1–1.3 mm	2.0 × 1.0 mm	2.7–3.1 × 1.8–2.1 mm

Note: The leaf data of *Impatiens huangyanensis* is our own independent measurements, which differ from the descriptions in the original literature.

### Phylogenetic relationship

**Figure 5.**
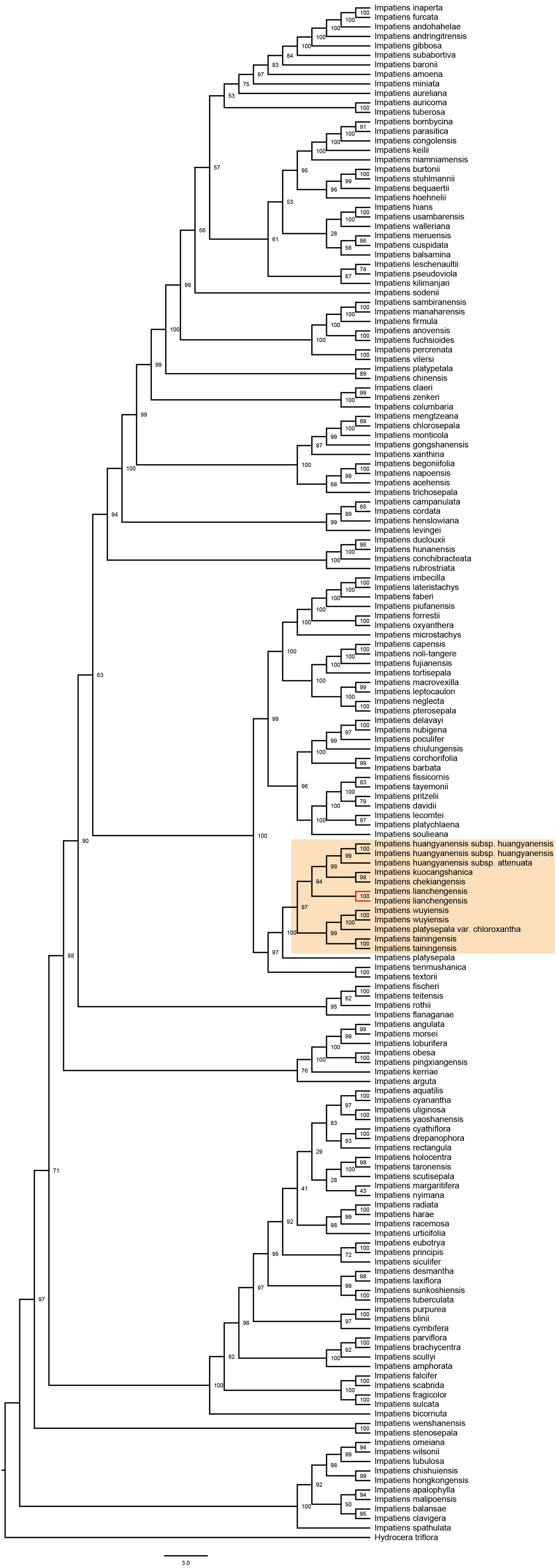
Phylogenetic tree based on ITS sequences. Numbers above branches are bootstrap support percentage values. *Impatiens lianchengensis* is shown in red.

**Figure 6.**
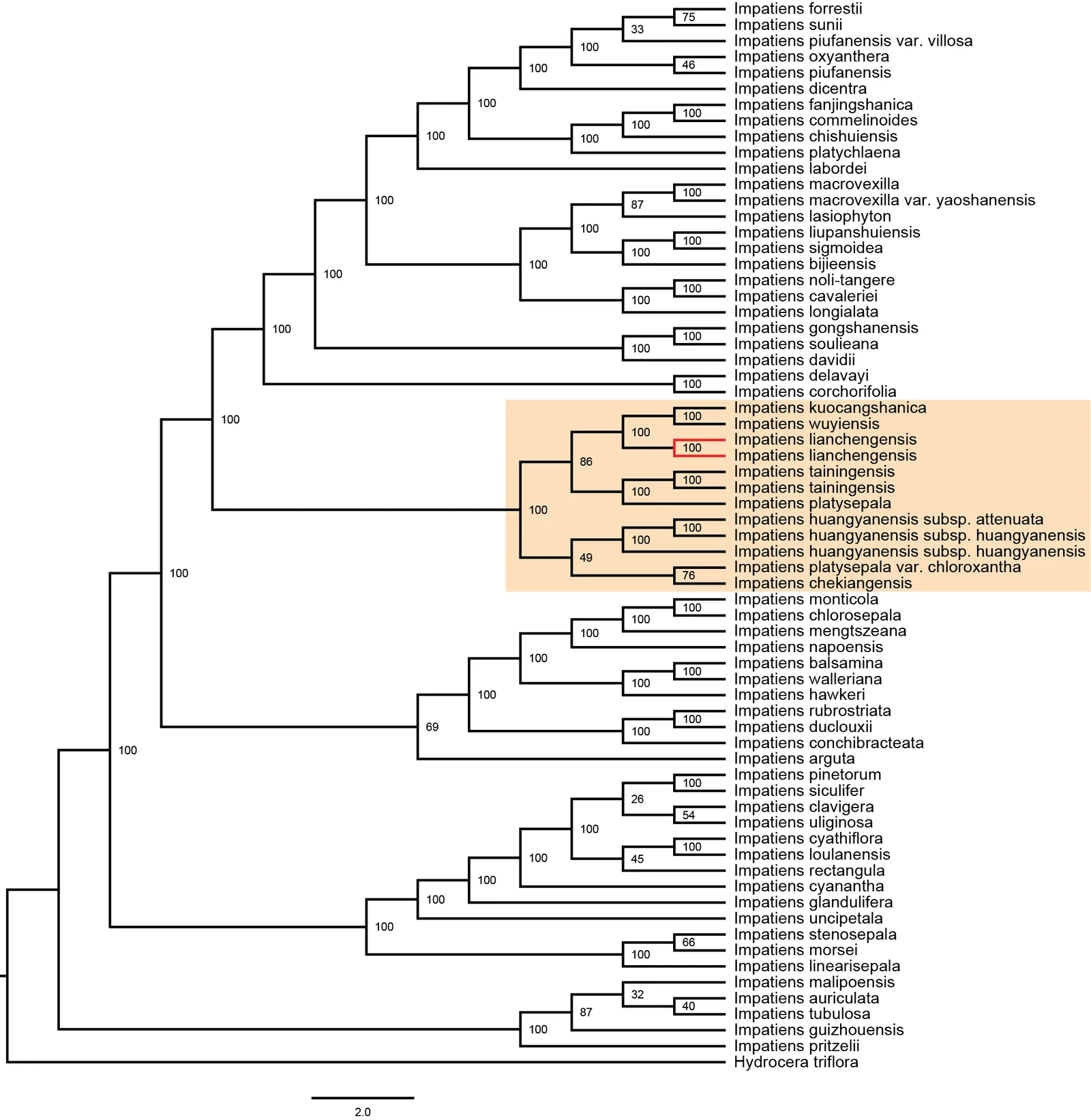
Phylogenetic tree based on the complete plastid genome sequence. Numbers above branches are bootstrap support percentage values. *Impatiens lianchengensis* is shown in red.

The alignment of the nuclear ribosomal ITS regions (ITS-1, 5.8S, and ITS-2) was 826 bp and included 162 species (Suppl. material 2). The phylogenetic tree based on ITS sequences indicated a close relationship between *Impatiens lianchengensis* and the clade of *I. huangyanensis*, *I. huangyanensis* subsp. *attenuata*, *I. kuocangshanica*, and *I. chekiangensis* (Fig. 5). The alignment of the plastid genome was 169,720 bp and included 65 species. Due to the lack of a complete plastome for *I. fujianensis*, it was not included in the analysis of the *Impatiens* plastid phylogeny. The results based on the complete plastid genome showed that *I. lianchengensis* clustered with *I. kuocangshanica* and *I. wuyiensis* as a highly supported clade (bootstrap 100%, Fig. 6). All results supported the new species status of *I. lianchengensis*.

### Characteristics of plastome

**Figure 7.**
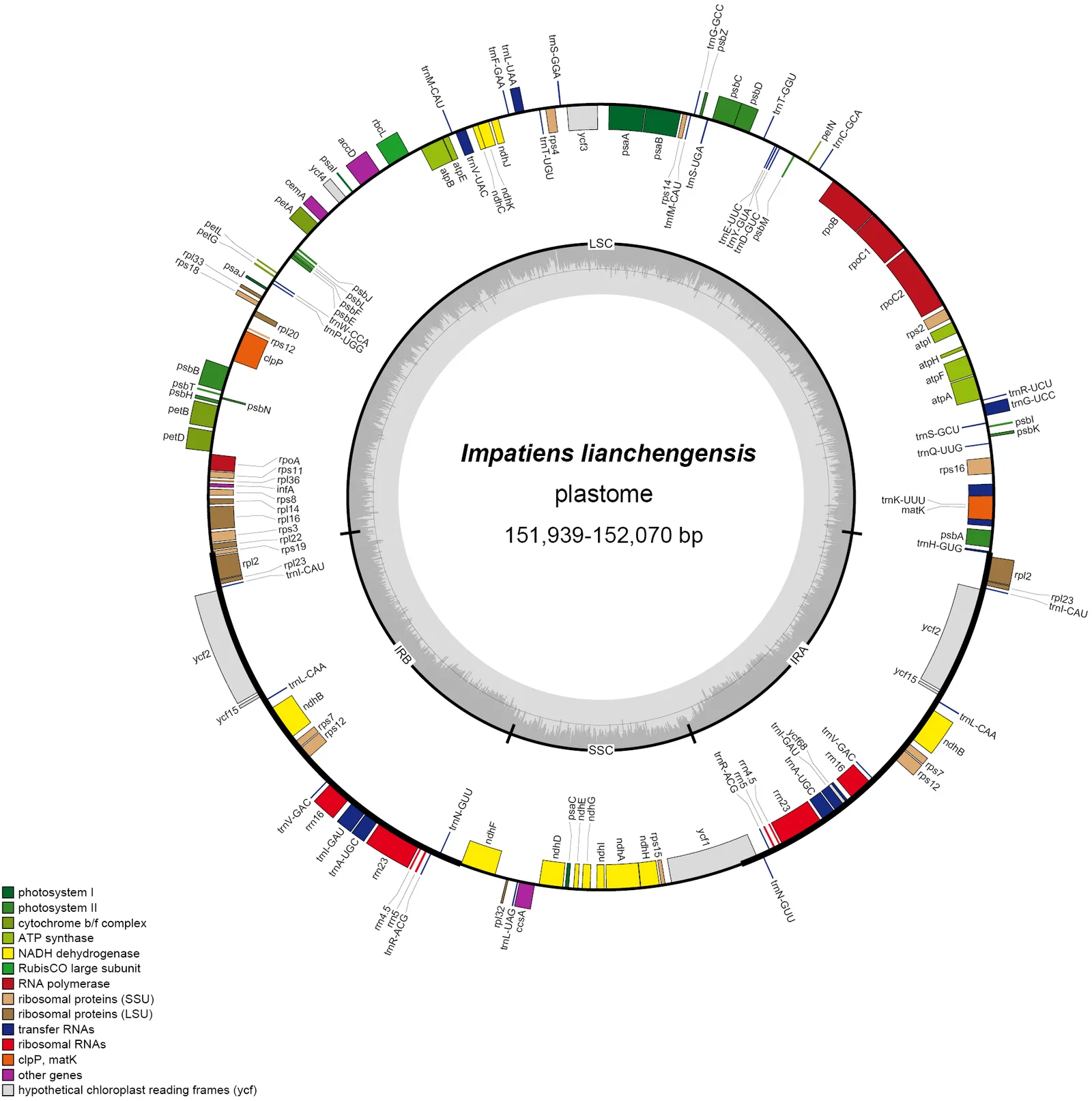
Plastome map of *Impatiens lianchengensis*. The inner dark gray circle corresponds to GC content, and the inner light gray circle corresponds to AT content. Different colors represent distinct genes within separate functional groups.

The complete plastome of *Impatiens lianchengensis* ranges from 151,939 to 152,070 bp in length and exhibits the typical quadripartite structure, comprising a large single-copy (LSC) region of 82,740–82,870 bp, a small single-copy (SSC) region of 17,680–17,685 bp, and a pair of inverted repeat (IR) regions of 25,757–25,760 bp each. The overall GC content is 36.8%, with corresponding values of 34.4–34.5% for the LSC, 29.3–29.4% for the SSC, and 43.1% for the IR regions. The plastome encodes a total of 134 genes, including 89 protein-coding genes, eight rRNA genes, and 37 tRNA genes. Among them, 17 genes are duplicated in the IR regions—six protein-coding genes (*ndhB*, *rpl2*, *rpl23*, *rps7*, *ycf2*, and *ycf15*), seven tRNA genes (*trnV*-GAC, *trnR*-ACG, *trnN*-GUU, *trnL*-CAA, *trnI*-CAU, *trnI*-GAU, and *trnA*-UGC), and all four rRNA genes (*rrn16*, *rrn23*, *rrn4.5*, and *rrn5*). The *rps12* gene occurs in three copies. In total, 10 protein-coding genes (*rps12*, *rps16*, *rpoC1*, *atpF*, *petB*, *petD*, *rpl16*, *rpl2*, *ndhB*, and *ndhA*) contain a single intron, while *ycf3* and *clpP* each contain two introns (Fig. 7).

### Taxonomic treatment of the new species

#### Impatiens lianchengensis

Taxon classificationPlantaeEricalesBalsaminaceae

J.D.Lin & P.Li sp. nov.

BB661E36-63E0-5A99-B20D-A369A4CD1F02

urn:lsid:ipni.org:names:77412431-1

Figs 1, 2

##### Type.

China • Fujian: Liancheng County, Guanzhaishan, moist places in the valley, 432 m; 25.7167, 116.8000; 6 May 2012; Fl., *Jiedong Lin*, *J. D. Lin 1201* (holotype: ZM; isotype: CSH, HZU, KUN, PE, FNU).

##### Diagnosis.

*Impatiens lianchengensis* resembles *I. huangyanensis* and *I. tainingensis* in the gross morphology of its pinkish purple flowers but differs by having obliquely ovate lateral sepals, a widely saccate lower sepal with a relatively wide mouth and distinctly attenuate posterior end, a shorter incurved spur (5.6–13.0 mm) apart at the bottom. It is further distinguished from *I. tainingensis* by the wider, more abruptly saccate lower sepal and shorter spur (5.6–13.0 mm vs. 15–18 mm) and from *I. huangyanensis* by the suborbicular upper petal (vs. oblate), obliquely ovate lateral sepals (vs. suborbicular), and smaller seeds (1.8–2.2 × 1.1–1.3 mm vs. 2.7–3.1 × 1.8–2.1 mm).

##### Description.

Herbs, annual, 30–70 cm tall, glabrous. ***Roots*** fibrous. ***Stem*** erect or basally ascending, robust and fleshy, simple or laxly branched, basal nodes swollen. ***Leaves*** alternate; petiole 2–4 cm long; blade papery, pale green, ovate-elliptic, 5–14 × 2–5 cm, lateral veins 5–8 pairs, curved, base cuneate, attenuate into petiole, margin crenate, teeth mucronulate, apex acuminate or caudate-acuminate; ***Inflorescences*** axillary, flowers 1–5; peduncles 1–2 cm long; pedicels 2–3 cm long, bracteate at base; bracts persistent, linear-lanceolate, 2–3 mm long. ***Flowers*** pinkish purple, ca. 3 cm; lateral sepals two, obliquely ovate, 7.9–11.4 × 5.7–10.1 mm, membranous, margin entire, apex acuminate; lower sepal purple striate, 12.2–23.4 mm deep, widely saccate, mouth relatively wide, posterior end distinctly attenuate, abruptly narrowed into an incurved spur apart at the bottom; spur 5.6–13.0 mm long; mouth vertical, 13.2–18.7 mm wide; upper petal suborbicular, 10.8–13.7 × 12.1–16.5 mm, apex emarginate, short rostellate, abaxial midvein cristate; lateral united petals 19.4–24.4 mm long, short clawed, 2-lobed; basal lobes obovate to dolabriform, small, 10.9–12.7 × 4.5–6.9 mm, apex obtuse; distal lobes dolabriform, large, 19.4–24.4 × 9.5–14.2 mm, apex obtuse or emarginate; auricle inflexed, yellow; filaments linear, anthers ovoid, apex obtuse; ovary erect, fusiform, ca. 5 mm long. ***Capsule*** fusiform or linear-cylindric, 16.7–25.0 mm long; seeds many, yellowish brown, ovoid-oblong, 1.8–2.2 × 1.1–1.3 mm, tuberculate.

##### Phenology.

Flowering and fruiting from May to September.

##### Distribution and habitat.

**Figure 8.**
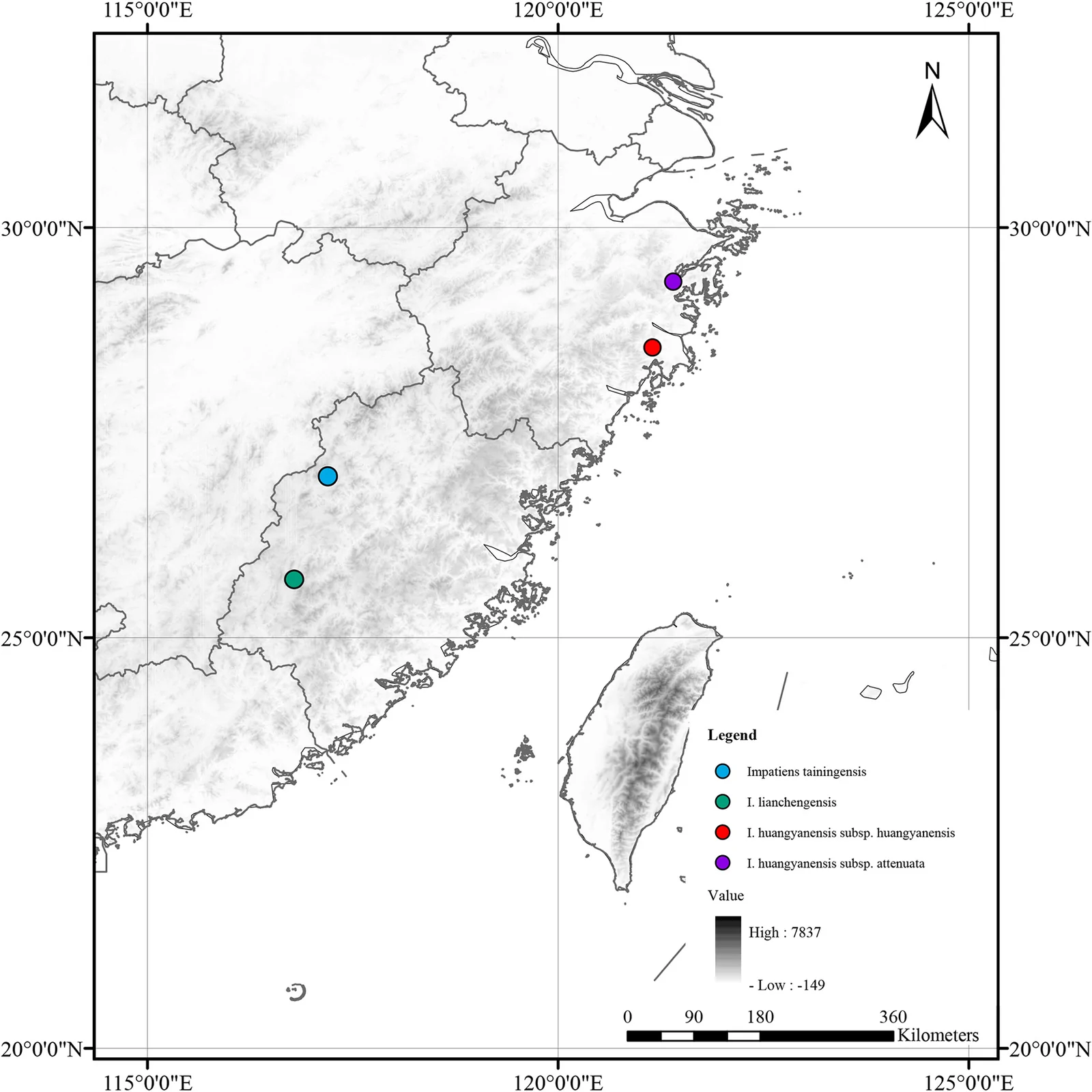
Distribution map of *Impatiens lianchengensis*, *I. tainingensis*, I. huangyanensis subsp. huangyanensis, and I. huangyanensis subsp. attenuata.

This new species is known from Liancheng County, western Fujian Province, China (Fig. 8). To date, it has been recorded at one locality in Liancheng County and grows in moist places in valleys at 400–500 m.

##### Etymology.

*Impatiens lianchengensis* was discovered on Mt. Guanzhai, Liancheng County, Fujian Province, China, from which the epithet *liancheng* is derived. Hence, the Chinese name ‘连城凤仙花 (lian cheng feng xian hua)’ is proposed.

##### Conservation status.

*Impatiens lianchengensis* is currently known only from Mt. Guanzhai Scenic Area, Liancheng County, Fujian Province. The two confirmed records fall within a single 2 × 2 km grid cell, giving an estimated area of occupancy (AOO) of 4 km^2^; the extent of occurrence (EOO) could not be reliably estimated from the available records. Fewer than 100 individuals were observed during the recent survey in August 2026, and several previously occupied sites no longer supported detectable individuals, suggesting a local decline. Although similar Danxia habitats nearby remain insufficiently surveyed, the known population represents a single location and is potentially threatened by localized habitat disturbance. *I. lianchengensis* is therefore provisionally assessed as Vulnerable (VU D2) according to the IUCN Red List criteria (IUCN Standards and Petitions Committee 2022).

##### Additional specimens seen (paratypes).

China • Fujian Province: Liancheng County, Guanzhaishan, 565 m, 25.71221°N, 116.78815°E, 30 Jun 2022, *Xinjie Jin, Junke Li & Jiedong Lin* Jin Xin Jie 347.

## Discussion

The integrative morphological and molecular evidence supports the recognition of *Impatiens lianchengensis* as a distinct species. Morphological comparisons among *Impatiens lianchengensis*, *I. huangyanensis*, and *I. tainingensis* are summarized in Table 1. Although *Impatiens lianchengensis* (Figs 1, 2) resembles *I. huangyanensis* and *I. tainingensis* in the gross morphology of its pinkish purple flowers, it can be distinguished by a combination of leaf and floral characters (Fig. 3). *I. lianchengensis* is characterized by obliquely ovate lateral sepals, a widely saccate lower sepal with a relatively wide mouth and distinctly attenuate posterior end, a shorter incurved spur apart at the bottom, and smaller seeds. It differs from *I. tainingensis* mainly in the shape of the lower sepal and its shorter spur and from *I. huangyanensis* in spur length, upper petal height and width, seed size, and the shapes of the lower sepal, lateral sepals, and upper petal. Notably, the measured ranges of upper petal height and width and seed length and width are non-overlapping between *I. lianchengensis* and *I. huangyanensis*. These characters, together with variation in plant height, leaf size, and inflorescence features, provide the principal morphological evidence for distinguishing *I. lianchengensis* (Fig. 4). The phylogenetically related species can also be readily distinguished from *I. lianchengensis* by floral morphology (Fig. 3). The flowers of *I. lianchengensis* are light pinkish purple, and its lower sepal is widely saccate, with a relatively wide mouth and a distinctly attenuate posterior end. In contrast, *I. kuocangshanica* has a funnelform lower sepal tapering into a long terminal spur. *I. wuyiensis* has entirely yellow flowers and a saccate-funnelform lower sepal tapering into a long spur (Wang et al. 2020). *I. chekiangensis* has pinkish-purple flowers but differs in having a narrowly funnelform lower sepal tapering into a long spur; its basal lobes of the lateral united petals are small and obovate, whereas the distal lobes are broadly obovate, larger, and emarginate at the apex, with a minute yellow auricle. *I. fujianensis*, another species occurring in Fujian Province, is readily distinguished by its pale yellowish-green flowers (Ma et al. 2024). These morphological differences further support the distinctiveness of *I. lianchengensis* from its phylogenetically related species.

The nuclear ITS and plastome phylogenies differ in some internal relationships within this group, but both analyses recover the two accessions of *Impatiens lianchengensis* as a distinct and strongly supported monophyletic lineage (Figs 5, 6). In the ITS tree, *I. lianchengensis* is placed within a clade containing *I. huangyanensis*, *I. huangyanensis* subsp. *attenuata*, *I. kuocangshanica*, *I. chekiangensis*, *I. wuyiensis*, and other related taxa, while remaining distinct from the *I. huangyanensis* lineage. In the plastome tree, the two accessions of *I. lianchengensis* form a strongly supported lineage within a subclade containing *I. wuyiensis* and *I. kuocangshanica*, whereas *I. huangyanensis* and *I. huangyanensis* subsp. *attenuata* occur in a separate subclade. Thus, despite differences in the internal topology of the nuclear and plastid trees, neither dataset supports the phylogenetic placement of *I. lianchengensis* within *I. huangyanensis*. The observed cytonuclear discordance may reflect limited phylogenetic signal in the ITS region or evolutionary processes such as incomplete lineage sorting, historical hybridization, or introgression (Qiu et al. 2023; Wang et al. 2026). These alternatives cannot be distinguished with the present data, and additional multilocus nuclear or population-genomic analyses will be required to resolve the relationships within this species complex more robustly.

The currently known distributions of *Impatiens lianchengensis* and *I. huangyanensis* are also geographically well separated (Fig. 8). *I. lianchengensis* is currently known from Mt. Guanzhai in western Fujian, whereas the confirmed locality of *I. huangyanensis* is in eastern Zhejiang. *I. huangyanensis* subsp. *attenuata* occurs still farther north in Zhejiang. This allopatric pattern is consistent with geographic differentiation among these taxa, although unsurveyed suitable habitats between the known localities mean that additional populations cannot yet be excluded. Geographic separation is therefore considered supporting evidence rather than evidence of reproductive isolation. The occurrence of several narrowly distributed species, including *I. lianchengensis*, *I. tainingensis*, *I. wuyiensis*, and *I. fujianensis*, in the Fujian Danxia region suggests that the heterogeneous and fragmented landscapes of southeastern China may have promoted localized radiation and diversification of *Impatiens*. However, the evolutionary processes underlying this pattern remain unclear. Further population-genomic, biogeographic, and ecological studies with broader geographic sampling will be necessary to test the roles of geographic isolation, historical gene flow, and habitat differentiation in the diversification of this group.

## Supplementary Material

XML Treatment for Impatiens lianchengensis

## Additional information

### Artificial Intelligence (AI) use

The authors accept full responsibility for the content of the manuscript, including the disclosure of any use of AI.

No AI tools were used in the preparation of this manuscript.

### Author ORCIDs

J. Lin https://orcid.org/0000-0002-2966-1561

J. Li https://orcid.org/0009-0004-7694-032X

B. Li https://orcid.org/0009-0005-8847-6883

X. Jin https://orcid.org/0000-0003-3733-1957

M. Wang https://orcid.org/0000-0002-3794-465X

P. Li https://orcid.org/0000-0002-9407-7740

### Data availability

The molecular data that support the findings of this study are openly available in GenBank (see Suppl. material 2).

## Supplementary materials

Supplementary material 1 Sampling information for *Impatiens lianchengensis* Data type csv https://binary.pensoft.net/file/1811055This dataset is made available under the Open Database License (http://opendatacommons.org/licenses/odbl/1.0/). The Open Database License (ODbL) is a license agreement intended to allow users to freely share, modify, and use this Dataset while maintaining this same freedom for others, provided that the original source and author(s) are credited. Jiedong Lin, Junlong Li, Boheng Li, Xinjie Jin, Meizhen Wang, Pan Li

Supplementary material 2 Species and GenBank accession numbers for the sequences used in this study Data type csv https://binary.pensoft.net/file/1811056This dataset is made available under the Open Database License (http://opendatacommons.org/licenses/odbl/1.0/). The Open Database License (ODbL) is a license agreement intended to allow users to freely share, modify, and use this Dataset while maintaining this same freedom for others, provided that the original source and author(s) are credited. Jiedong Lin, Junlong Li, Boheng Li, Xinjie Jin, Meizhen Wang, Pan Li
